# Asymptomatic Left Bundle Branch Block Predicts New-Onset Congestive Heart Failure and Death From Cardiovascular Diseases

**DOI:** 10.4021/cr214w

**Published:** 2012-11-20

**Authors:** Peyman N. Azadani, Ata Soleimanirahbar, Gregory M. Marcus, Thaddeus J. Haight, Milton Hollenberg, Jeffrey E. Olgin, Byron K. Lee

**Affiliations:** a Penn State Hershey Medical Center, PA, USA; b University of California, San Francisco, School of Medicine, San Francisco, CA, USA; c University of California, Berkeley, School of Public Health, Berkeley, CA, USA

**Keywords:** Bundle-branch block, Heart failure, Mortality, Risk factors

## Abstract

**Background:**

Left bundle branch block (LBBB) has been proposed as a risk factor for cardiovascular morbidity and mortality. We sought to characterize the strength of these associations in a population without preexisting clinical heart disease.

**Methods:**

The association between LBBB and new-onset congestive heart failure (CHF) or death from cardiovascular diseases was examined in 1,688 participants enrolled in the SPPARCS study who were free of known CHF or previous myocardial infarction. SPPARCS is a community-based cohort study in residents of Sonoma, California that are > 55 years. Medical history and 12-lead ECGs were obtained every 2 years for up to 6 years of follow-up. LBBB at enrollment or year 2 was considered “baseline” and assessed as a predictor of CHF and cardiovascular death ascertained at years 4 and 6.

**Results:**

The prevalence of LBBB at baseline was 2.5% (n = 42). During 6 years of follow-up, 70 (4.8%) people developed new CHF. Incidence of CHF was higher in patients with LBBB than in participants without LBBB. This association persisted after controlling for potential confounders (odds ratio (OR): 2.85; 95% confidence interval (CI): 1.01 - 8.02; P = 0.047). A higher mortality from cardiovascular diseases was also found in participants with LBBB after adjusting for potential confounders (OR: 2.35, 95%CI: 1.02 - 5.41; P = 0.044).

**Conclusions:**

LBBB in the absence of a clinically detectable heart disease is associated with new-onset CHF and death from cardiovascular diseases. Further study is warranted to determine if additional diagnostic testing or earlier treatment in patients with asymptomatic LBBB can decrease cardiovascular morbidity or mortality.

## Introduction

The electrocardiogram (ECG) is one of the most commonly ordered tests in everyday clinical practice. Consequently, detecting subjects with abnormalities of the cardiac conduction system such as left bundle branch block (LBBB) in the absence of clinically detectable heart disease is relatively common [1, 2].

It is not clear what cardiovascular morbidities to expect, if any, when asymptomatic patients develop LBBB. Previous studies investigating cardiac conduction abnormalities have focused mainly on survival of patients with LBBB in the setting of ischemic heart disease and acute coronary artery disease [3-7]. The significance of LBBB has not been well studied in subjects without myocardial infarction or preexisting heart failure. Therefore, we sought to characterize the long-term prognostic implication of asymptomatic LBBB on congestive heart failure (CHF) development and death from cardiovascular diseases in a large generally healthy population.

## Methods

We analyzed data from the Study of Physical Performance and Age-related Changes in Sonomans (SPPARCS) Project. The project is a community based prospective longitudinal cohort study of physical activity and physical fitness in people 55 years or older that live in Sonoma, CA or in the city’s vicinity. The methods of recruitment and the representative of the sample to its target population have been described [8]. A community-based census identified 3,057 age eligible individuals. Eventually 2,092 people enrolled in the study and completed the baseline interview. Among these participants, 1,688 people with no history of CHF or myocardial infarction were recruited for this study.

Enrolled participants underwent detailed standardized interviews and filled out standardized questionnaires. These included detailed questions about medical conditions. Identified medical conditions were ascertained by physician's diagnosis and medical treatment, 12 lead ECG testing was done at regular 2 year intervals. Patients were followed for 4 total assessments unless they died, left the study, or were lost to follow-up. The primary outcomes of the current study were incident CHF and death from cardiovascular diseases. Cardiovascular deaths were those included in International Classification of Disease 9th Revision (ICD9) codes 394 through 448. CHF and cardiovascular death were ascertained at the 3rd and 4th assessments.

LBBB was defined as (1) QRS duration > 120 ms, (2) PQ interval > 120 ms, (3) predominantly upright complexes with slurred R waves in leads I, V_5_, and V_6_, and (4) small of absent R waves in leads V_1_ and V_2_. The other predictors examined were demographic and medical characteristics including age, gender, atherosclerosis, hypertension, diabetes, kidney disease, and COPD at each time interval. Age was recorded as a continuous variable and all the other risk factors were recorded as a yes/no dichotomous variables.

The statistical analyses were performed using SPSS version 16. Categorical data were compared using the χ^2^ test, and continuous data were compared using ANOVA. Logistic regression analyses were used to determine the relationship of LBBB with CHF and cardiovascular death in both bivariate models and multivariable analysis controlling for all potential confounders. A value of P < 0.05 was considered to be statistically significant. The study was approved by the Committee on Human Research at the University of California, San Francisco and the Committee for protection of Human Subjects at the University of California, Berkeley. Written informed consent was obtained from all subjects in the study prior to data collection. No extramural funding was used to support this work. The authors are solely responsible for the design and conduct of this study; all study analyses, the drafting and editing of the paper and its final contents.

## Results

### Characterization of participants

**Figure 1:**
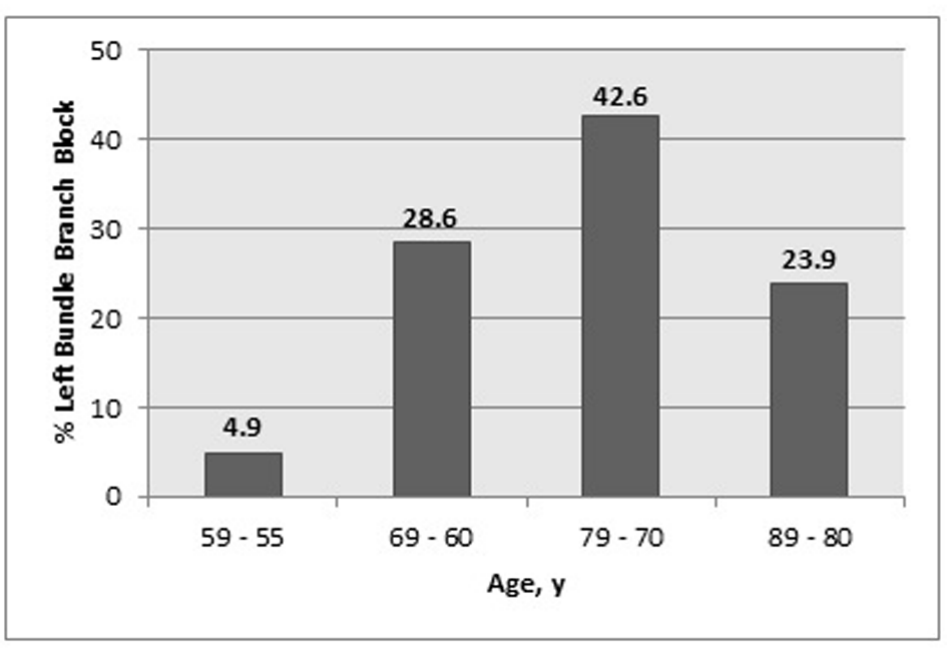
Age distribution of LBBB at baseline.

A total of 1,688 individuals aged 55 years or older were enrolled and followed for at least 6 years. Shown in Figure 1, the prevalence of LBBB at baseline was 2.5% (n = 42). Subjects with LBBB were older (mean age 74.0 ± 8.4 years vs. 69.2 ± 8.3 years, P < 0.001) and reported a greater prevalence of atherosclerosis (31.0% vs. 16.3%, P < 0.05). The baseline characteristics of both groups are shown in Table 1.

**Table 1:** Baseline Characteristic of the Study Population According to Their ECG LBBB Diagnosis

Characteristic	With LBBB (n = 42)	Without LBBB (n = 1,646)	Total (n = 1,688)
Male, n (%)	17 (40.5)	669 (40.6)	686 (40.6)
Age, y (mean ± SD)	74.0 ± 8.4*	69.2 ± 8.3	69.4 ± 8.3
Atherosclerosis, n (%)	13 (31.0)*	268 (16.3)	281 (16.6)
Hypertension, n (%)	23 (54.8)	720 (43.7)	743 (44.0)
Diabetes, n (%)	4 (9.5)	101 (6.1)	105 (6.2)
Kidney disease, n (%)	2 (4.8)	44 (2.7)	46 (2.7)
COPD, n (%)	2 (4.8)	194 (11.8)	196 (11.6)

* P < 0.05; COPD: Chronic obstructive pulmonary disease.

### Congestive heart failure development

During follow up, 70 (4.8%) people of the study population developed CHF. In bivariate analysis, baseline LBBB was associated with a 3.64 greater odds of developing CHF (95% confidence interval (CI): 1.37, 9.71; P = 0.006). In multivariable logistic regression analysis adjusting for potential confounders, participants with baseline LBBB remained nearly three (2.85) times more likely to develop CHF (Table 2). Other risk factors for the development of CHF identified in this model were age, atherosclerosis, diabetes, and COPD.

**Table 2:** Multivariable Logistic Analysis of LBBB as a Predictor of Incident CHF and Cardiovascular Death

Characteristics	Incident CHF Cumulative Incidence Ratio		Cardiovascular Death Cumulative Incidence Ratio	
	(95% CI)	P	(95% CI)	P
Demographic characteristics				
Age	1.06 (1.02 - 1.09)	0.001	1.15 (1.12 - 1.18)	0.000
Male gender	1.11 (0.67 - 1.85)	0.629	1.64 (1.11 - 2.44)	0.014
Medical/environmental history				
LBBB	2.85 (1.01 - 8.02)	0.047	2.35 (1.02 - 5.41)	0.044
Atherosclerosis	3.05 (1.79 - 5.19)	0.000	2.66 (1.75 - 4.03)	0.000
Hypertension	1.68 (0.99 - 2.84)	0.051	1.57 (1.05 - 2.35)	0.027
Diabetes	2.31 (1.09 - 4.91)	0.029	1.18 (0.59 - 2.37)	0.644
Kidney Disease	1.15 (0.26 - 5.18)	0.852	1.95 (0.73 - 5.18)	0.182
COPD	2.21 (1.14 - 4.26)	0.018	1.07 (0.60 - 1.88)	0.824

LBBB: Left bundle branch block; COPD: Chronic obstructive pulmonary disease; CHF: Congestive heart failure; CHF identified in this model were age, atherosclerosis, diabetes, and COPD.

### Mortality

**Figure 2:**
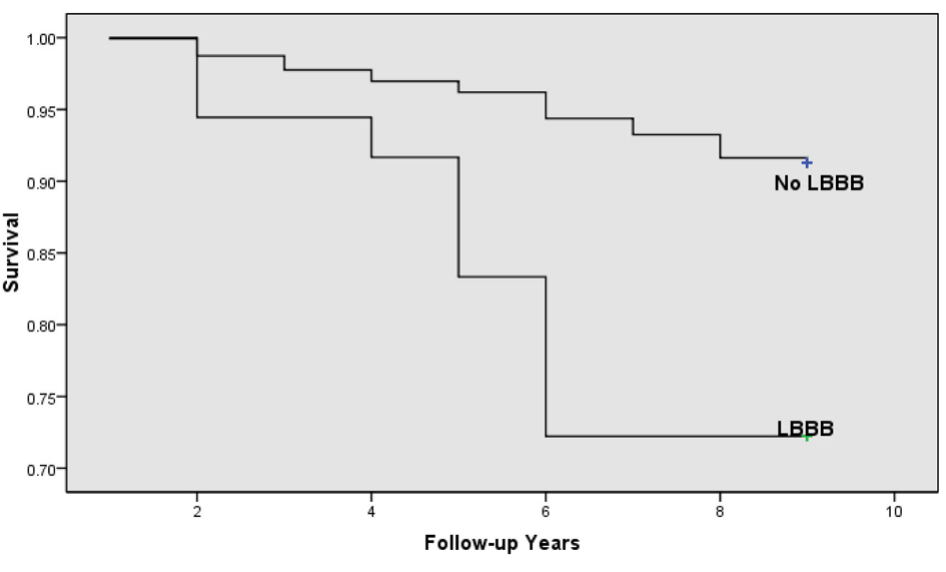
Kaplan-Meier survival curve showing the effects of left bundle branch block on cardiovascular mortality by age (P < 0.05).

Of a total of 351 (20.1%) deaths from all causes that occurred during follow-up, 133 (7.9%) were listed as cardiovascular diseases (Table 3). Unadjusted mortality rate from cardiovascular and cardiac diseases was higher among patients with LBBB compared to those without LBBB. In bivariate analysis, patients with LBBB had 4.34 times greater odds of dying from cardiovascular disease (95% CI: 2.02, 8.89; P = 0.001; Fig. 2). This association remained significant after controlling for other potential confounders (Table 2). Other independent risk factors for cardiovascular death in our population were age, gender, atherosclerosis, and hypertension.

**Table 3:** Causes of Death of the Study Population

Cause of death (n = 362)	Number (%)
Cardiovascular	134 (37.1)
Cardiac	97 (72.4)^a^
Atherosclerotic cardiovascular disease	14 (14.4)^a^
Cardiac arrest	2 (2.1)
Congestive heart failure	23 (23.7)
Coronary artery disease	7 (7.2)
Heart disease	51 (52.6)
Noncardiac	37 (27.6)
Stroke	34 (91.9)^a^
Other (aortic aneurysms)	3 (8.1)
Non-cardiovascular	228 (62.9)

^a^: Percent of category deaths.

## Discussion

Individuals with asymptomatic LBBB had an increased incidence of CHF and death from cardiovascular disease. It is unclear if LBBB is a marker of subclinical cardiac disease that manifests overtly later, or if LBBB is intrinsically deleterious, causing CHF and cardiovascular death.

The association between LBBB and cardiovascular morbidity has been investigated, but given conflicting results, controversy regarding the prognosis of LBBB persists. Fahy observed a higher rate of developing overt cardiovascular disease among people with isolated LBBB [2]. However, he did not specify the type of cardiovascular diseases. In the Framingham Study, newly acquired LBBB was associated with an advanced underlying cardiac abnormality including symptomatic hypertensive or ischemic heart disease [9]. On the other hand, Rotman did not observe any significant difference in incident cardiovascular morbidity among subjects with LBBB [10]. Notably, participants in that study were not free of cardiovascular disease. In a population based study, Erikson found a higher incidence of CHF among men with bundle branch block [11]. Notably, the studied population was not free of myocardial infarction or heart failure, women were not included, and this association was found in patients with any type of bundle branch block.

One might speculate on a possible causal role in which LBBB may lead to CHF. It is believed that right-to left ventricle endocardial activation that occurs in LBBB results in left ventricular dyssynchrony, compromising systolic and diastolic function [12]. This dyssynchrony may also trigger remodeling processes that lead to progressive left ventricular dysfunction [13]. Alternatively, bundle branch block has been shown to be associated with a degenerative process of both the conduction system and the myocardium itself which could lead to various dysrhythmias and conduction disturbances as well as impair myocardial function [11].

The association between LBBB and mortality has been previously suggested by a few studies in the literature [14, 15]. In community-based studies, all-cause and cardiovascular mortality was higher among patients with asymptomatic LBBB [2, 9, 14]. In this study, we looked at different classifications of cardiovascular death and the association of isolated LBBB and cardiovascular mortality in an older population free of any known previous CHF or myocardial infarction based on very detailed interviews and questionnaires. Our results suggest that LBBB, in the absence of overt clinical heart disease, is significantly associated with death from cardiovascular diseases. The nature of this association remains unclear. Whether LBBB is yet another marker that reflects a generalized physiological aging process (for example, diabetes) or is more directly and causally involved in the pathway to cardiovascular death remains unanswered. Although we controlled for cardiovascular disease risk factors including diabetes, coronary artery disease, and hypertension, the possibility that cardiovascular disease risk are more severe in patients with LBBB than in the normal population may account for the higher mortality rate found in patients with LBBB.

In view of our findings and those of others, it appears reasonable to question whether the finding of asymptomatic LBBB should prompt further diagnostic testing or early treatment that might prevent CHF and cardiovascular morbidity. Currently, neither further testing nor treatment is recommended when asymptomatic LBBB is found. Other studies would be helpful to evaluate possible therapeutic options. The benefit of early treatment of diagnosed CHF has been previously shown by other studies in patients following myocardial infarction [16]. Heart failure treatment may also prove to be beneficial in patients with asymptomatic LBBB who have yet to develop CHF, possibly by staving off the progression to symptomatic CHF.

### Limitations

Several limitations exist to our study. First, the assessment of medical conditions was based on interviews with the patient rather than independent adjudication. For example, participants may have been unaware of underlying CHF or may have failed to recollect the diagnosis when completing the baseline questionnaire. Second, our assessment of LBBB was based on one to two 12 lead ECGs done over 2 years. Therefore, some participants may have had intermittent LBBB that was not detected. However, this likely would have resulted in decreased power and should not have resulted in false positive associations.

### Conclusions

Our data indicate that patients with asymptomatic LBBB are at increased risk of developing CHF and of dying from cardiovascular diseases. Further study is warranted to determine if more diagnostic testing or earlier treatment in patients with asymptomatic LBBB can decrease cardiovascular morbidity or mortality.
